# Pitfalls for laparoscopic pancreaticoduodenectomy: Need for a stepwise approach

**DOI:** 10.1002/ags3.12242

**Published:** 2019-03-12

**Authors:** Jonathan Geograpo Navarro, Chang Moo Kang

**Affiliations:** ^1^ Division of Surgical Oncology Department of Surgery Vicente Sotto Memorial Medical Center Cebu City Philippines; ^2^ Division of HBP Surgery Department of Surgery Yonsei University College of Medicine Seoul Korea; ^3^ Pancreatobiliary Cancer Center Yonsei Cancer Center Severance Hospital Seoul Korea

**Keywords:** laparoscopic pancreaticoduodenectomy, pitfall, step‐wise approach, technique

## Abstract

Because of today's advancements in surgical techniques and perioperative management skills, surgeons are beginning to explore the usefulness of the laparoscopic approach in managing periampullary tumors. However, as a result of its innate complexity and associated high surgery‐related complications, its applicability to the general surgical community remains controversial. To date, only retrospective data from high‐volume centers support the safety and feasibility of laparoscopic pancreaticoduodenectomy (Lap PD) for the treatment of benign conditions and malignant periampullary tumors. In addition, various surgical techniques in terms of port placement, dissection, and reconstruction have evolved in different centers depending on the preferred method commonly used by the surgeon through accumulated experience. In our center, we used a stepwise approach and standardized our surgical technique to overcome this technically demanding procedure. A collaborative implementation of video review and analysis, practice training and simulation, operating room didactics, and strict adherence to our stepwise approach in Lap PD, might potentially improve the surgical skills of young hepatobiliary surgeons and possibly overcome the volume‐based learning curve of Lap PD.

## 1 INTRODUCTION

Pancreaticoduodenectomy is one of the most challenging surgical procedures for most surgeons to carry out. In particular, for malignant periampullary tumor, the radicality of the resection itself warrants careful tissue handling to preserve the oncological benefit. As such, open pancreaticoduodenectomy remains the standard approach for a periampullary tumor.

However, in the era of minimally invasive surgery, surgeons are beginning to explore the usefulness and safety of laparoscopic pancreaticoduodenectomy (Lap PD). Pioneered by Gagner and Pomp in 1994 in a patient with pancreatic divisum,^1^ its application in the management of periampullary tumors has subsequently spread worldwide.^2^, ^3^ Although there have been no randomized controlled studies, accumulated experience and retrospective data support the safety and effectiveness of Lap PD in treating benign conditions and malignant periampullary tumors.^3^, ^4^ However, until a well‐designed prospective randomized controlled trial validates its feasibility and safety, the potential benefit of Lap PD for malignant and benign tumors will remain uncertain. Its innate technical complexity and the potential risk of severe surgery‐related complications might hinder its general applicability.

Nevertheless, with today's advancements in surgical techniques and perioperative management skills, Lap PD is thought to be an appropriate approach for treating periampullary pathological conditions, particularly in high‐volume centers.^5^ With proper selection criteria and wise intraoperative decision‐making for open conversion, Lap PD can be safely implemented in daily clinical practice.

Most importantly, standardization of various surgical techniques might be the key to overcome their innate complexity. Therefore, we describe our surgical technique and apply a stepwise approach as a benchmark to overcome the pitfalls of Lap PD.

## 2 INDICATIONS FOR LAP‐PD

Surgical indications for Lap PD may be similar to those for Open PD.^6^, ^7^, ^8^ Benign and low‐grade malignant (borderline malignant) periampullary tumors are the best indications for Lap PD when considering long‐term survival and quality of life, as minimal or no lymphadenectomy is needed.^9^ Cases with periampullary cancers should be selected cautiously, because, in general, Lap PD may be technically very difficult in patients with severe obesity, severe cholangitis, pancreatitis, and potential risk of combined major vascular resection. Particularly when dealing with mid‐bile duct cancer, resection‐margin problems can occur during the operation, which make Lap PD more difficult, require longer operation time to obtain an additional margin for curative resection, and possibly decrease oncological benefit. However, it should be emphasized that surgeon expertise and experience in Lap PD can influence whether the patient undergoes the procedure. For instance, vascular resection can be carried out in a high‐volume center with well‐experienced surgeons.^10^, ^11^, ^12^, ^13^ For these reasons, patients with comorbidities and in poor general condition who cannot tolerate a lengthy pneumoperitoneum would find it difficult to undergo Lap PD.

**Figure 1:**
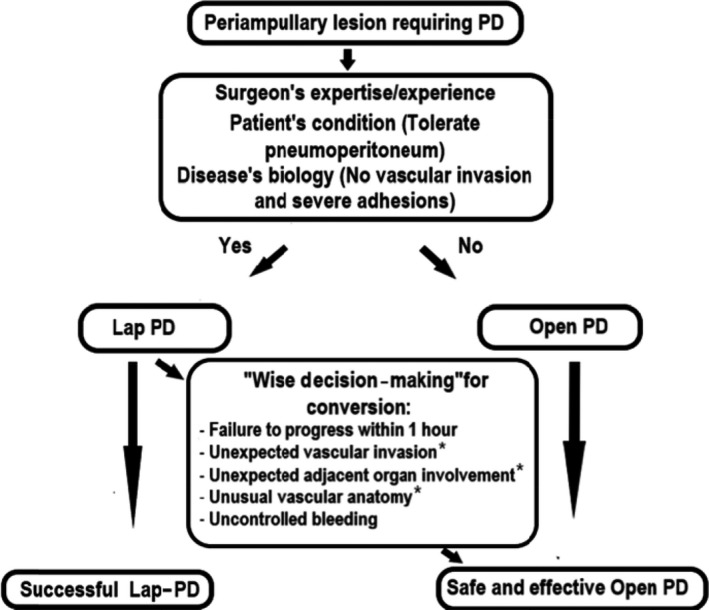
Indications and basis for selection of laparoscopic pancreaticoduodenectomy (Lap PD). *Only expert surgeon can carry out this level of difficulty during Lap PD

Nevertheless, we standardized our Lap PD selection criteria for patients with periampullary tumor. Whether to carry out Lap PD or Open PD can be decided preoperatively or intraoperatively. In general, we select patients with benign and low‐grade malignant periampullary lesions, periampullary cancer not involving major vascular structure, and good performance status as good candidates for Lap PD. Intraoperatively, wise decision‐making for open conversion to laparotomy is paramount. For patient safety, failure to progress within 1 hour of dissection as a result of severe pancreatitis or adhesions, unexpected vascular invasion, unexpected adjacent organ involvement requiring combined organ resection, unusual vascular anatomy, and uncontrolled bleeding may warrant open conversion to laparotomy.^6^ Although combined segmental resection of the major venous system is feasible and safe,^14^ only expert surgeons can carry out this difficult intraoperative situation to complete a safe and successful Lap PD. Figure 1 illustrates the indications and basis for selection of Lap PD.

## 3 EXTENT OF SURGICAL DISSECTION

Standard dissection during PD is thought to be optimal when carrying out Lap PD.

There are several important randomized controlled studies comparing extended and standard dissection in periampullary cancer. Most of the studies concluded that there was no survival benefit from carrying out extended lymphadenectomy compared to standard lymphadenectomy.^15^, ^16^, ^17^ Recently, Jang et al^18^ reported on a Korean multicenter collaborative study and concluded that there was no survival advantage in carrying out extended PD compared to standard PD. In their study, standard resection included dissection of lymph node stations 12b, 12c, 13, and 17 without nerve dissection around the hepatic artery or superior mesenteric artery (SMA), whereas extended resection included removal of lymph node stations 8, 9, 13, 17, 12, 14, and 16. Moreover, the nerve plexus or ganglion on the right side of the celiac axis and SMA was dissected semicircumferentially. These findings are the same as the results from a multicenter randomized controlled trial from Japan that failed to show survival benefits in patients undergoing extended PD compared to standard PD.^19^

Therefore, based on currently available evidence and surgical techniques of Lap PD, standard dissection is feasible when carrying out Lap PD. However, extended dissection including para‐aortic lymph nodes and perineural soft tissue around the SMA is allowed for margin‐negative resection in selected cases and by selected expertise.

## 4 PORT PLACEMENT AND OPERATING ROOM SETTING

**Figure 2:**
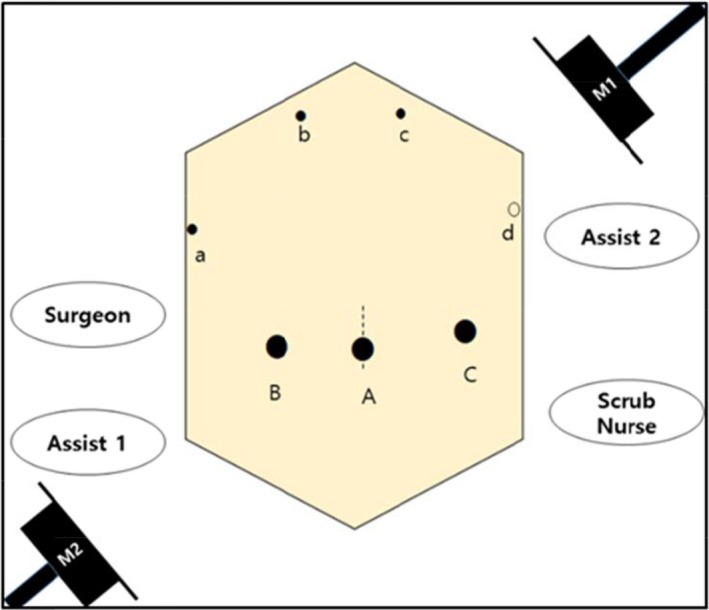
Placement of trocars and operator position. The surgeon stands at the right side of the patient during the entire surgical procedure. The laparoscopic camera is usually introduced through right‐sided 12‐mm ports (A and B) according to the target surgical field by Assist 1. Port a and port A (or B) will be the main working ports for the surgical procedure. Assist 2 can help to expose the surgical field using Ports b, c, and C. Port d can be added in case of robotic reconstruction. Two monitors (M1 and M2) are placed on the opposite side of the surgeons and nurses for ergonomic surgical interventions

Placement and number of trocars varied across centers. The number of trocars varied from five to seven.^20^, ^21^, ^22^ Depending on the surgeon's preference, it is most important that the surgeon can manipulate the instruments ergonomically and achieve the best exposure. Our approach in Yonsei, however, used six to seven trocars, as presented in Figure 2.

### 4.1 Devices

Three laparoscopic graspers, two laparoscopic dissectors, and one laparoscopic needle holder with straight plastic handles were used for Lap PD. Ultrasonic (recently referred to as a vessel‐sealing system) and bipolar energy devices are fundamental requirements for Lap PD. A bipolar energy device is especially useful in blunt dissection and for complete coagulation around major vascular structures.

## 5 STEPWISE APPROACH TO LAPAROSCOPIC PANCREATICODUODENECTOMY

Different surgical techniques in terms of port placement, dissection, and reconstruction have been reported in different high‐volume centers.^21^, ^23^, ^24^, ^25^, ^26^, ^27^, ^28^, ^29^ As such, standardization of an operative technique may vary across regions or countries depending on the prepared method commonly used by the surgeon. However, it should be emphasized that implementation of a standard protocol is paramount to overcome the volume‐based learning curve for Lap PD.^30^, ^31^ Moreover, innovative training strategies should also be implemented for training young surgeons in the field of pancreatic surgery.^32^

Recently, we began to explore the feasibility of laparoscopic pancreaticoduodenectomy for treating periampullary tumor and began to standardize our surgical technique.^14^, ^33^, ^34^, ^35^ In our center, to augment hepatobiliary surgical training, we strictly implemented video review and analysis, practice training and simulation, operating room didactics, and strict implementation of our stepwise approach in Lap PD.

In a stepwise approach, Lap PD consists of the “resection phase” and the “reconstruction phase.” Each is subdivided into a series of steps to guide the beginner's approach to Lap PD (Table 1).

**Table 1:** Stepwise approach for laparoscopic pancreaticoduodenectomy

Resection stage	
Step 1	Division of the gastrocolic ligament
Step 2	Control of the right gastrocolic vessels
Step 3	Control of the right gastric artery
Step 4	Transection of the first portion of the duodenum
Step 5	Dissection of the superior border of the pancreatic neck
Step 6	Dissection of the inferior border of the pancreatic neck
Step 7	Creation of the pancreatic window and taping
Step 8	Dissection of the hepatoduodenal ligament and common hepatic duct taping
Step 9	Dissection of the pancreatic head and colonic mesentery
Step 10	Mobilization of the duodenum and pancreatic head and division of the ligament of Treitz
Step 11	Division of the proximal jejunum and control of mesenteric vessels
Step 12	Division of the pancreas and uncinate process dissection
Step 13	Division of the bile duct
Reconstruction stage	
Step 1	Pancreaticojejunostomy anastomosis
Step 2	Hepaticojejunostomy anastomosis
Step 3	Extracorporeal duodenojejunostomy

According to tissue condition, the operation sequence may be changed, but the step‐by‐step approach in Table 1 will guide beginners through a safe and effective Lap PD.

### 5.1 Laparoscopic resection stages

#### 5.1.1 Division of the gastrocolic ligament

**Figure 3:**
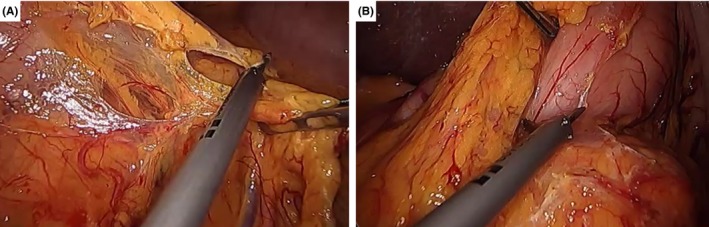
(A) Dissection of the gastrocolic ligament up to the (B) pyloric area

An incision is made into the gastrocolic ligament a few centimeters from the gastroepiploic arcade of the body of the stomach, using a LigaSure instrument (Medronics Inc, Boulder, Colorado, USA). By doing this, the left‐hand grasper of the operator grasps the stomach while the assistant left‐hand grasper retracts the colonic side to create an effective tension for dissection (Figure 3). The dissection will be continued up to the left gastroepiploic artery arcade. In this area, carrying out the procedure becomes much easier when the assisting surgeon lifts the stomach, because the surgeon can then use two hands for effective dissection. The gastrocolic ligament is further divided along the same plane toward the pyloric area to expose the right gastroepiploic vessels.

#### 5.1.2 Control of the right gastroepiploic vessels

**Figure 4:**
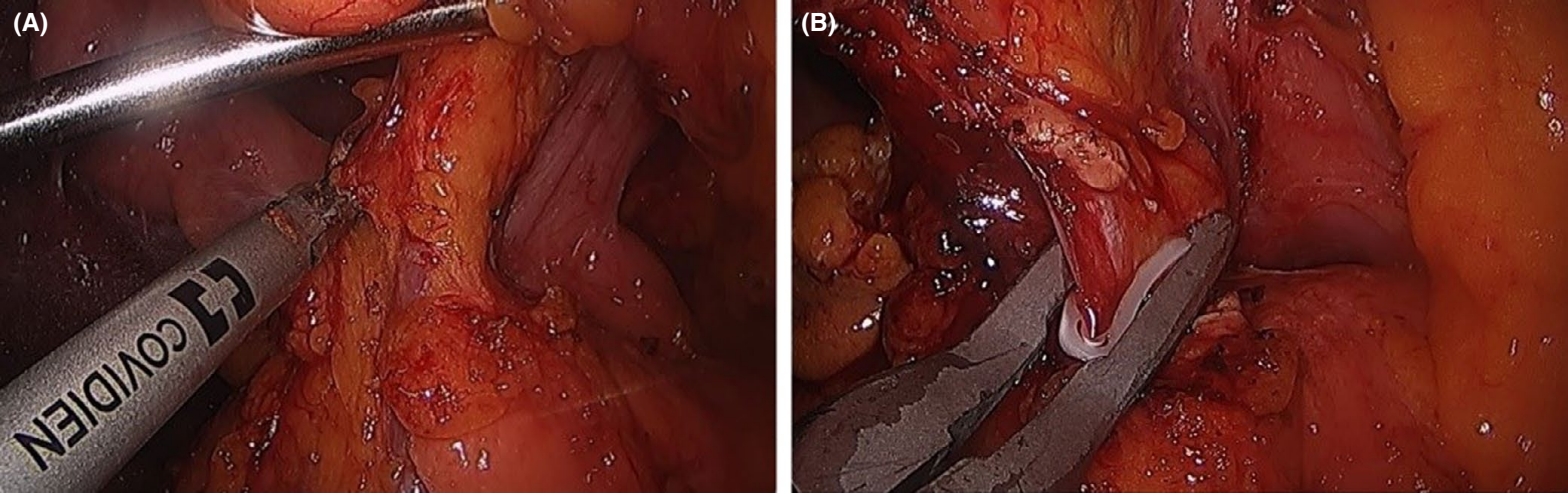
(A) Dissection and (B) ligation of the right gastroepiploic vessels

The assistant lifts the stomach in a 12 o'clock position to properly expose the pyloric area. This will clearly expose the right gastroepiploic vessels, which can be effectively ligated using laparoscopic clips. Either individual control of the artery and the vein or simultaneous control of these vessels is acceptable. This will also facilitate dissection and separation of the antero‐superior part of the head of the pancreas and the inferior part of the first portion of the duodenum (Figure 4). Bleeding is usually encountered in this area and can be effectively controlled using laparoscopic clips and a bipolar energy device. When dissecting and controlling the right gastroepiploic vessels, removing too many of them from the stomach will result in vascular insufficiency around the duodenal stump, which would prevent a duodenojejunostomy.

#### 5.1.3 Control of the right gastric vessels

**Figure 5:**
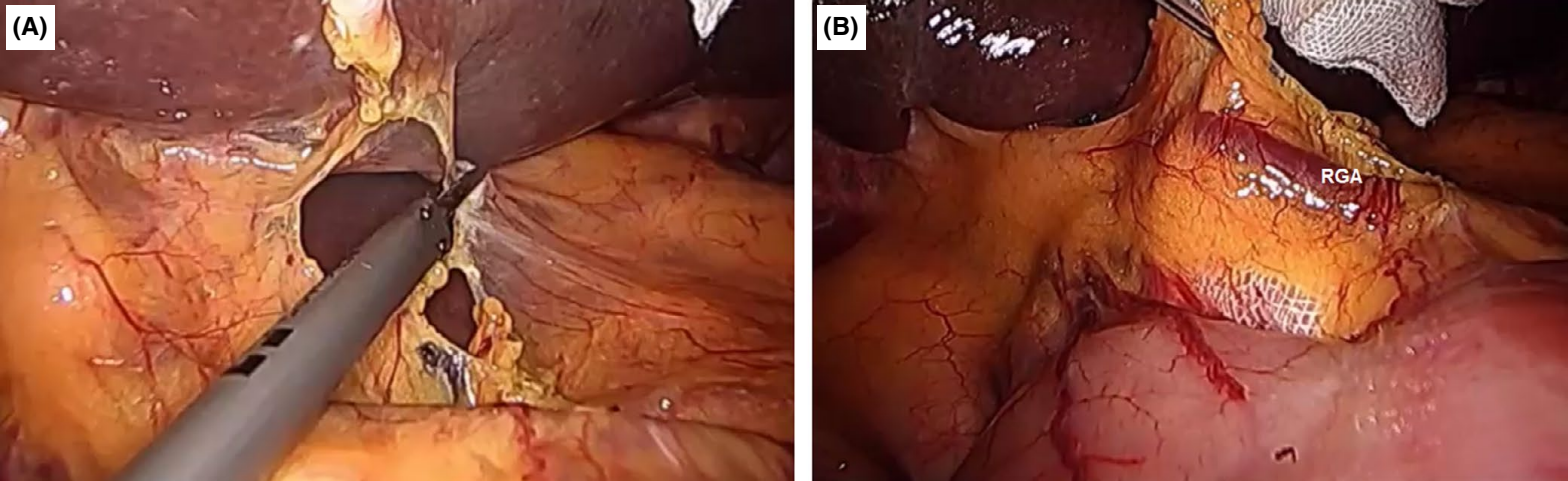
(A) Dissection of the hepatogastric ligament exposes the (B) right gastric artery (RGA)

Next, the superior part of the first portion of the duodenum is dissected. The assistant right‐hand grasper retracts the liver while the assistant left‐hand grasper retracts the first portion of the duodenum to expose the hepatoduodenal ligament. The peritoneal lining of the hepatoduodenal ligament is incised just above the first portion of the duodenal wall and along the lesser curvature of the stomach antrum to expose the right gastric artery (RGA, Figure 5). Once identified, it can be secured with laparoscopic clips and transected. For procedural efficacy, origin of the RGA should not be identified at that time. Instead, it can be identified and controlled during the stage of hepatoduodenal ligament dissection. In some cases, the left hepatic artery from the left gastric artery runs within the lesser sac. This vessel needs to be preserved in case of unexpected potential complications requiring total embolization of the hepatic artery as a result of gastroduodenal artery stump bleeding.

#### 5.1.4 Division of the duodenum

**Figure 6:**
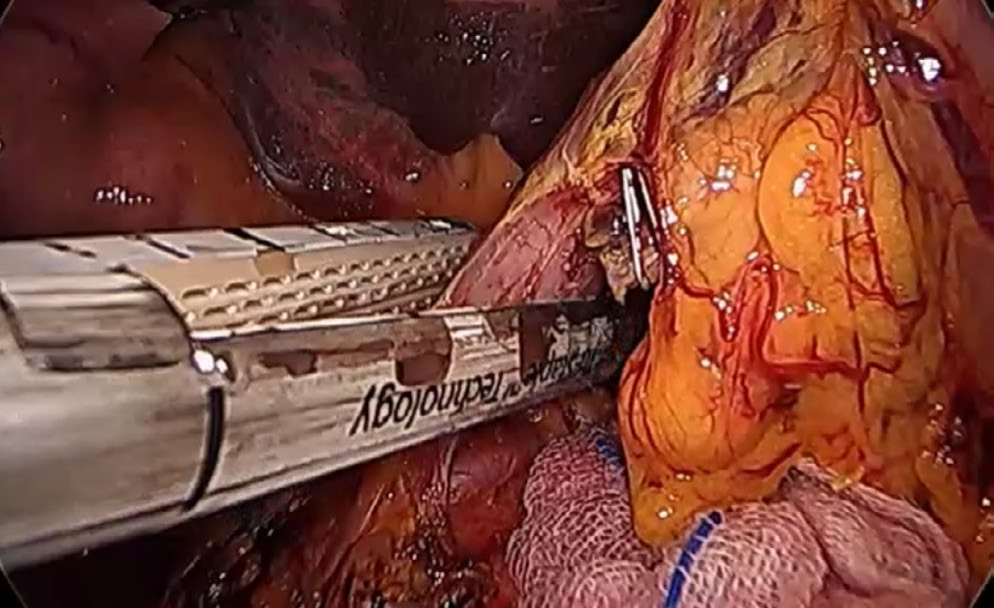
Division of the first portion of the duodenum using an Endo‐GIA stapling device (Medronics Inc, North Haven, CT, USA)

After transection of the RGA, the first portion of the duodenum can be freed from the hepatoduodenal ligament attachment. At this time, complete mobilization of the first portion of the duodenum can be achieved. The left‐hand grasper of the assistant will hold the inferior portion of the stomach antrum to position the first portion of the duodenum in an oblique position toward the left side of the patient. An Endo‐GIA stapler (Medronics Inc, North Haven, CT, USA) is inserted and positioned from the greater curvature to the lesser curvature 1‐2 cm from the pylorus (Figure 6). The transected stomach is then placed in the left subdiaphragmatic area.

#### 5.1.5 Dissection of the superior border of the pancreatic neck

**Figure 7:**
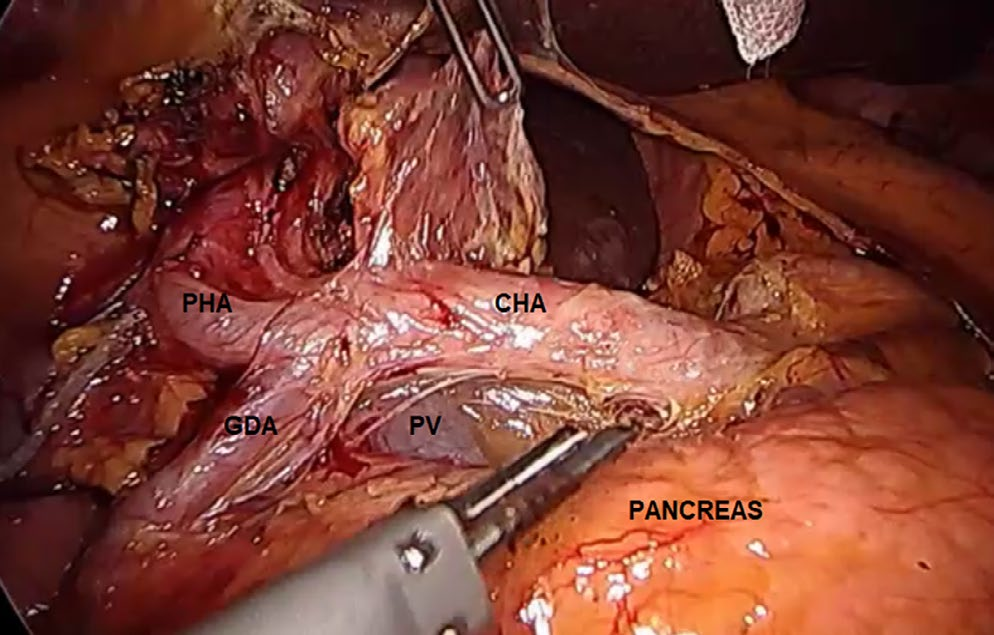
Dissection of the superior border of the pancreas. The portal vein (PV) can be exposed and identified by dissecting the triangle bordered by the superior border of the pancreas inferiorly and by the gastroduodenal artery (GDA) and common hepatic artery (CHA) superolaterally. PHA, proper hepatic artery

The left‐hand grasper of the assistant will pull down the mesenteric attachment of the inferior border of the pancreas to allow a clear view of the superior border of the pancreas. The peritoneal lining of the hepatoduodenal ligament is incised along the pancreatic neck. Once opened, the common hepatic artery is usually identified and can be followed caudally to expose the gastroduodenal artery. The portal vein can be identified and exposed by dissecting the triangular‐shaped tissue bordered by the common hepatic artery, gastroduodenal artery, and superior border of the neck of the pancreas (Figure 7). This can be facilitated by coagulation and blunt dissection of the fatty tissues around this area using a bipolar energy device.

#### 5.1.6 Dissection of the inferior border of the pancreatic neck

**Figure 8:**
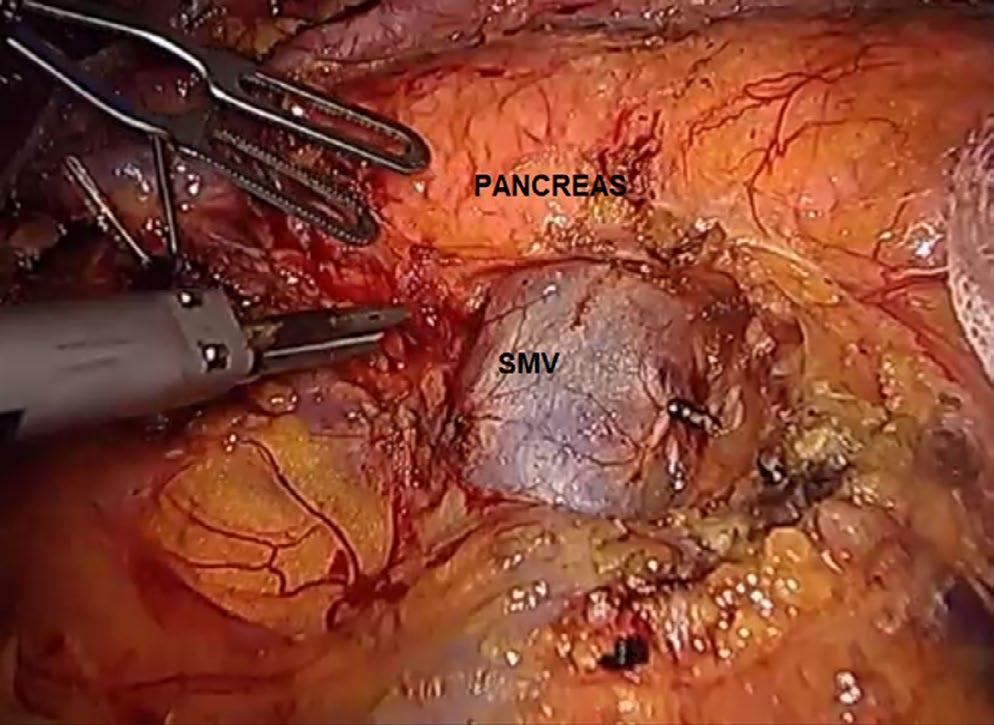
Dissection of the inferior border of the pancreas. SMV, superior mesenteric vein

The transected right gastroepiploic vein can be followed down to its origin to identify the location of the superior mesenteric vein (SMV). Once the SMV is identified, the left‐hand grasper of the assistant holds the right dorsal part of the mesentery just caudal to the origin of the right gastroepiploic vein on the SMV. By doing this, the SMV can be clearly exposed for further dissection. The peritoneal lining on the antero‐inferior aspect of the neck of the pancreas is dissected along the adventitia of the SMV, clearing all fatty tissues around it up to the inferior border of the pancreas (Figure 8). Small tributaries of the SMV might be encountered during dissection and can be safely secured using laparoscopic clips.

#### 5.1.7 Pancreatic neck window and taping

**Figure 9:**
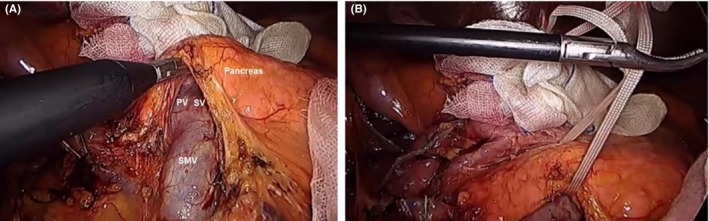
A, Creation of a pancreatic window exposing the portal vein (PV) and splenic vein (SV). B, Completed taping of the pancreas. SMV, superior mesenteric vein

Once the dissection reaches the inferior border of the pancreas, the inferior peritoneal covering is dissected toward the left and right sides of the SMV. Using blunt dissection with a bipolar energy device, a small window can be created between the anterior border of the SMV and the posterior neck of the pancreas. A large right‐angled dissector can now be inserted along this window up to the superior border of the neck of the pancreas (Figure 9). Once the dissector visualizes the superior border of the pancreas, a tape is placed around the neck of the pancreas to facilitate later transection.

#### 5.1.8 Dissection of the hepatoduodenal ligament and bile duct taping

**Figure 10:**
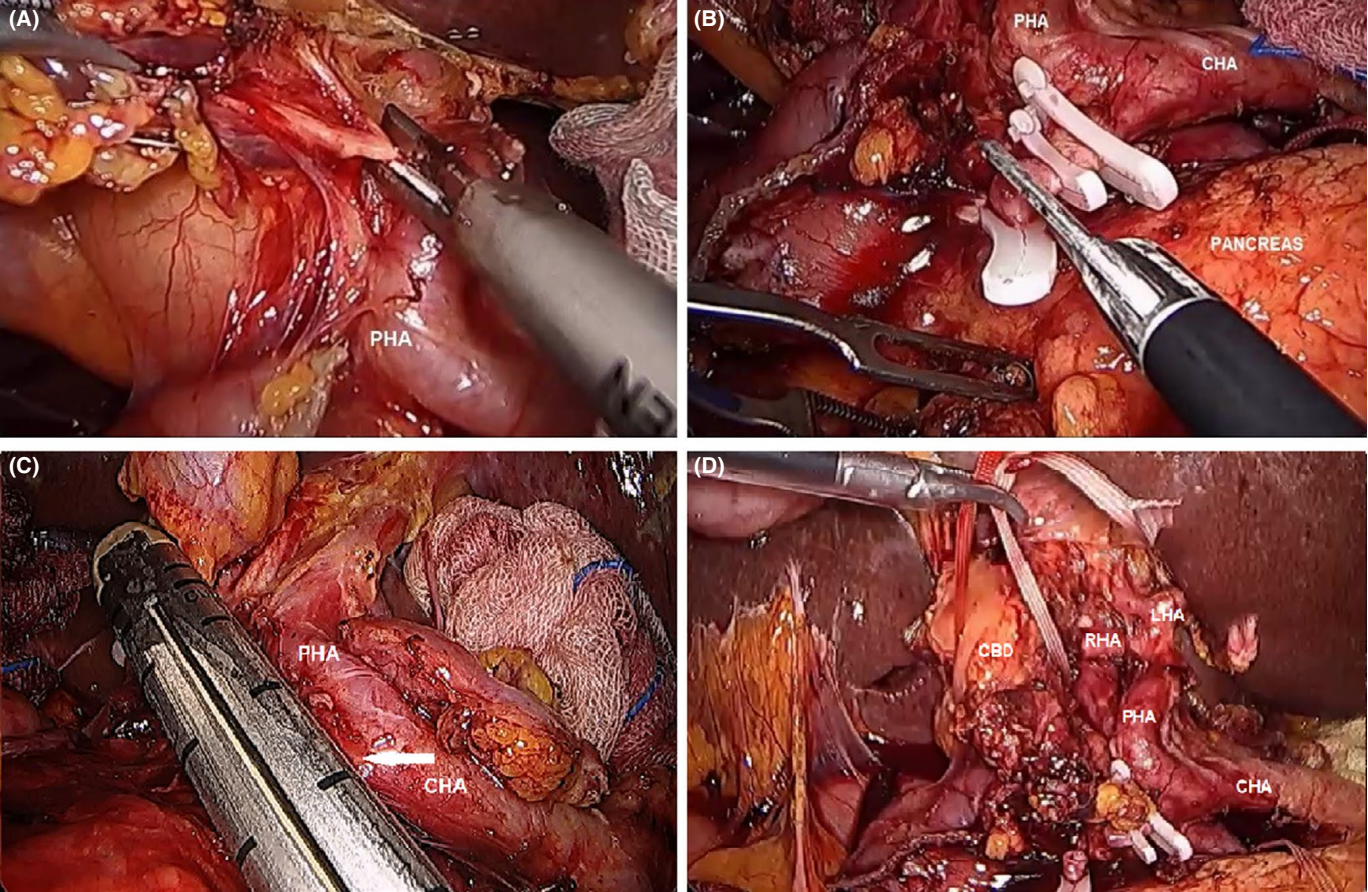
A, Dissection of the hepatoduodenal ligament follows the route of the hepatic arteries. B, The gastroduodenal artery is ligated and transected to facilitate exposure of the portal vein. C, In some cases, the gastroduodenal artery (white arrow) can be secured using an Endo‐GIA stapler (Medronics Inc, North Haven, CT, USA). D, A tape is encircled around the common hepatic duct. CBD, common bile duct; CHA, common hepatic artery; LHA, left hepatic artery; PHA, proper hepatic artery; RHA, right hepatic artery

During this time, the right‐hand grasper of the assistant holds the ligamentum teres to facilitate liver retraction. Dissection of the hepatoduodenal ligament (HDL) is started at the junction of the common hepatic artery and gastroduodenal artery, removing all the lymph nodes and connective tissues surrounding the common hepatic artery and superior margin of the pancreas (Figure 10A). Dissection can be easily facilitated using energy devices. At this time, the gastroduodenal artery is controlled with laparoscopic clips or Endo‐GIA (Figure 10B,C). Dissection is continued along the proper hepatic artery toward the left and right hepatic arteries. In some cases associated with severe cholangitis, indocyanine green (ICG) technology will allow easy identification of the common bile duct (CBD). Dissection of the CBD is started by dissecting Calot's triangle and carrying out ligation of the cystic artery and duct. The cystic duct can be used as a guide and traction of the CBD during dissection. The CBD is then dissected, separated from the portal vein, and circled and secured by tape (Figure 10D).

#### 5.1.9 Dissection of the pancreatic head and colonic mesentery (control of the gastrocolic trunk)

**Figure 11:**
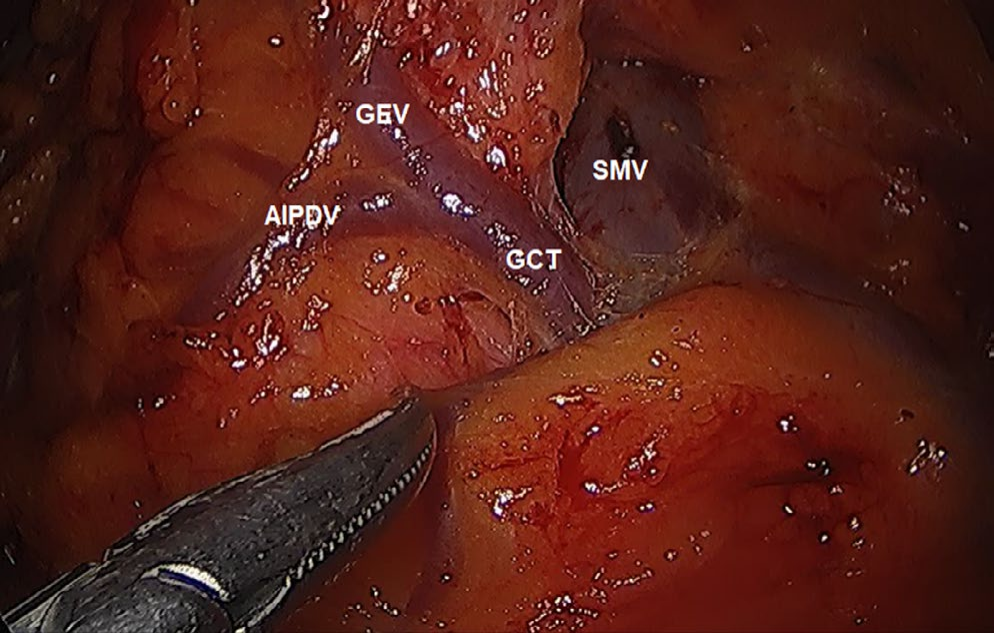
Gastrocolic trunk (GCT). AIPDV, anterior inferior pancreaticoduodenal vein; GEV, gastroepiploic vein; SMV, superior mesenteric vein

Proper orientation of the head and body of the pancreas is one of the key points during this procedure. The avascular plane of the anterior aspect of the transverse mesocolon is followed to the head of the pancreas. During this time, the gastrocolic trunk can be visualized within the mesentery. The transected right gastroepiploic vein (RGEV) can be followed down to its origin in the gastrocolic trunk. The anterior inferior pancreaticoduodenal vein on the right lateral side of the RGEV can also be identified to its origin in the gastrocolic trunk (Figure 11). With a combination of blunt and sharp dissection, the gastrocolic trunk is followed up to the SMV, which can be safely ligated with laparoscopic clips.

#### 5.1.10 Mobilization of the duodenum and pancreatic head and division of the ligament of Treitz

**Figure 12:**
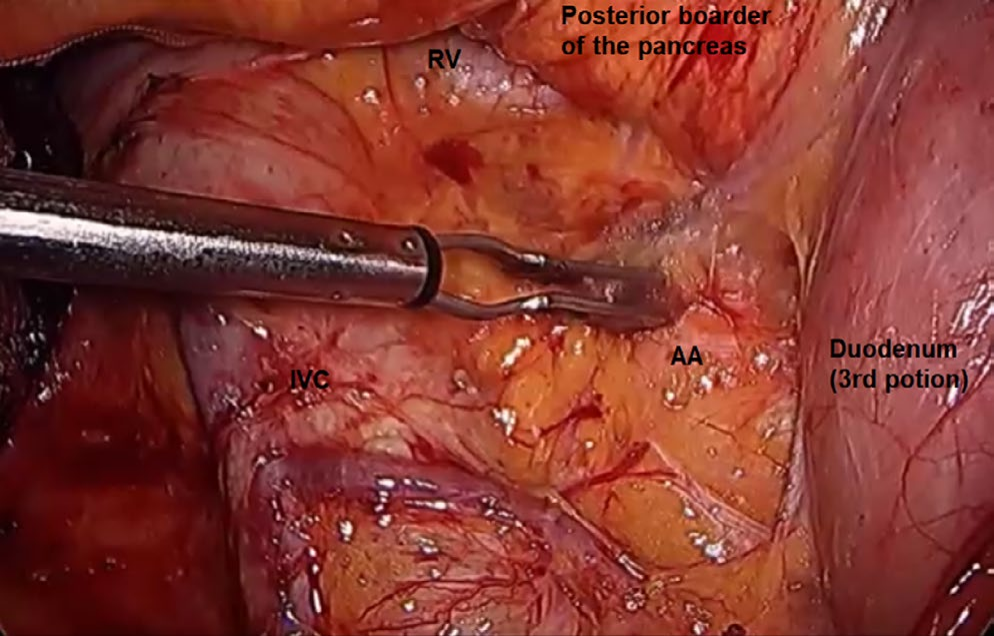
Full kocherization. AA, abdominal aorta; IVC, inferior vena cava; RV; renal vein

A full Kocher's maneuver was carried out to mobilize the duodenum and head of the pancreas. The lateral peritoneal attachments of the duodenum were dissected, exposing the inferior vena and aorta. Dissection was continued to the lateral border of the aorta (Figure 12). The assistant right‐hand grasper retracted the second portion of the duodenum toward the left side, while the left‐hand grasper retracted the right lateral side of the transverse mesocolon for wide exposure of the C‐loop of the duodenum. Care must be taken as the duodenum is easy to tear, leading to bile spillage into the abdominal cavity during the procedure.

**Figure 13:**
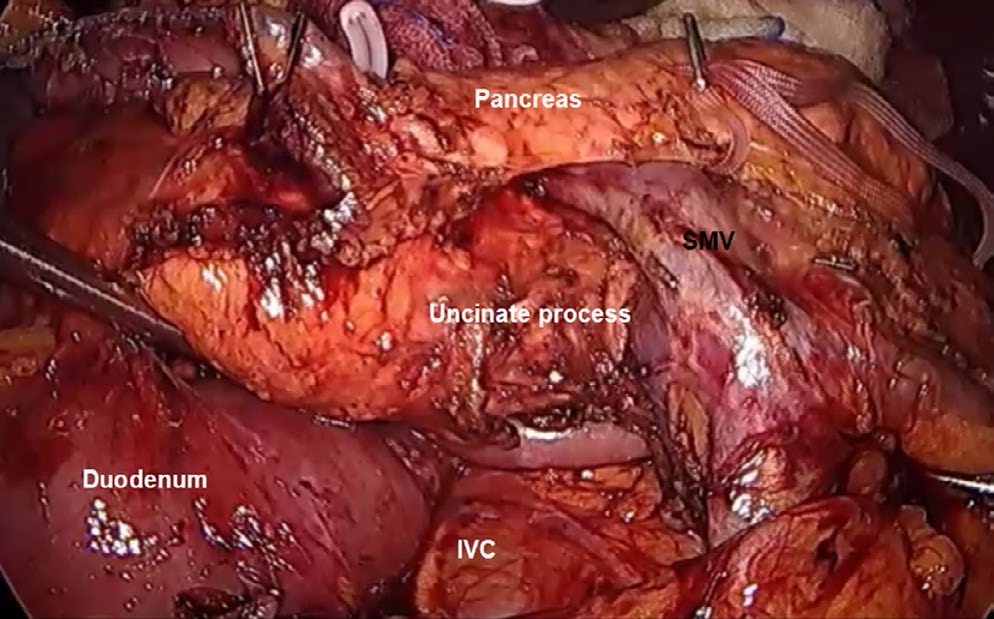
Image after complete mobilization of the duodenum and pancreatic head. IVC, inferior vena cava; SMV, superior mesenteric vein

Once full kocherization is achieved, separation of the duodenum and head of the pancreas from the colonic mesentery can start through dissection along the right lateral border of the SMV. Once the SMV is identified, the left‐hand grasper of the assistant holds the right dorsal part of the mesentery just caudal to the origin of the right gastroepiploic vein on the SMV and pulls it left to rotate the mesentery in a clockwise position. When this is done, the SMV can be clearly exposed for further dissection. Dissection will continue along the adventitia of the SMV, clearing all fatty tissues around it up to the inferior border of the uncinate process pancreas (Figure 13). Small tributaries of the SMV might be encountered during dissection and can be safely secured using laparoscopic clips.

##### Para‐aortic lymph node sampling

**Figure 14:**
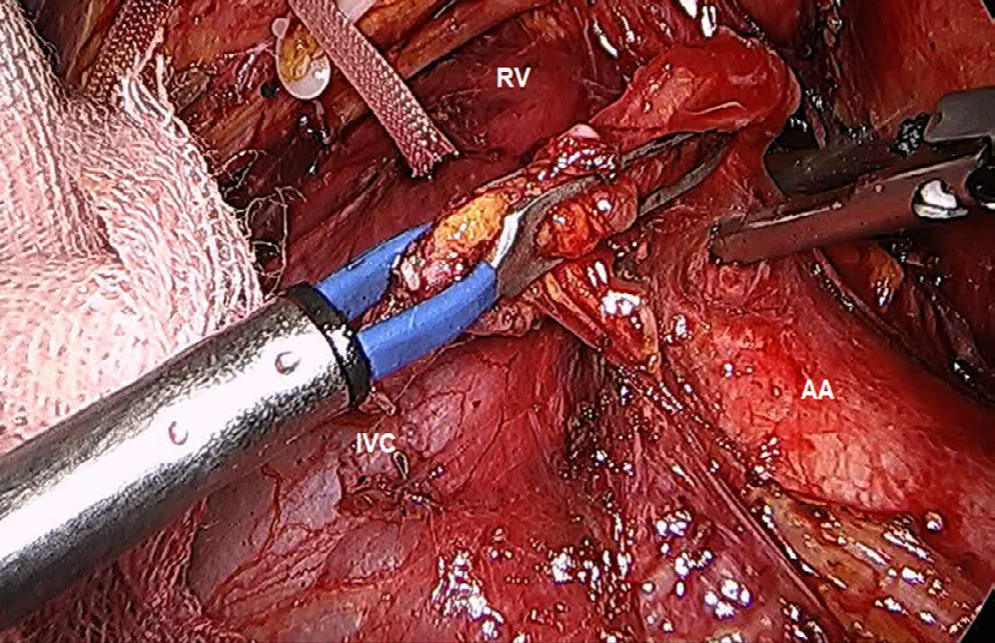
Para‐aortic lymph node sampling. AA, abdominal aorta; RV; renal vein

In cases where there were some enlarged lymph nodes around the para‐aortic area, the lymph nodes and some connective tissues anterior to the inferior vena cava and aortocaval (station 16a and 16b) area were sampled for frozen section biopsy (Figure 14).

#### 5.1.11 Division of the proximal jejunum and control of mesenteric vessels

**Figure 15:**
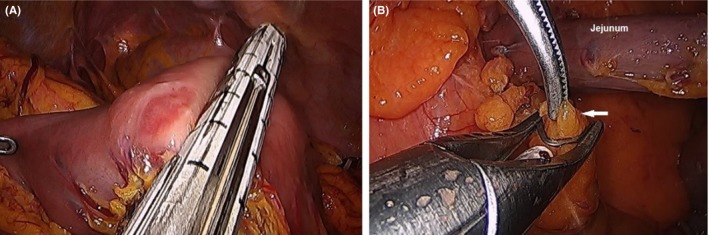
A, Division of the proximal jejunum. B, Ligation of mesenteric vessels (white arrow) using a LIGACLIP (Aesculap Incorporated, Center Valley, PA, USA)

The right‐hand grasper of the assistant holds the transverse colon and retracts it cephalad to expose the ligament of Treitz. The proximal 10‐cm segment of the jejunum is identified. The length of the proximal jejunum can vary according to patient's condition. For example, obese patients have a longer proximal jejunum, which needs to be considered for future retrocolic fashioned pancreaticojejunostomy and hepaticojejunostomy in the reconstruction phase. The assistant left‐hand grasper holds the distal part of the jejunum while the surgeon holds the proximal part. The mesentery is dissected down to its mesenteric root. The jejunum is then transected using an Endo‐GIA (Figure 15A). The mesentery of the proximal jejunum is pulled down using a LigaSure close to the mesenteric border of the jejunum, and the mesenteric vessels are then clipped to avoid postoperative bleeding (Figure 15B). The ligament of Treitz is pulled down, and the mesenteric attachments and small vessels of the third and fourth portions of the duodenum are dissected and clipped. The duodenum and jejunal segment are then exposed underneath the mesentery and placed on the right side.

#### 5.1.12 Pancreas division and uncinate process dissection

**Figure 16:**
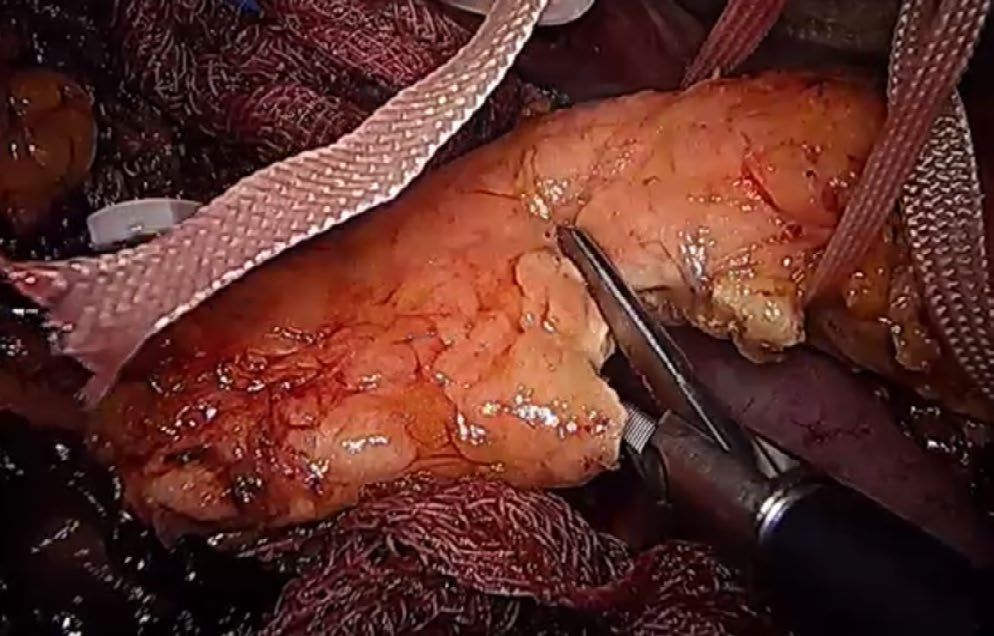
Transection of the pancreatic neck

Next, the tape around the neck of the pancreas is pulled up at a 12 o'clock position. An energy device is used to partially transect the pancreas up to its duct, after which the transection is continued using scissors (Figure 16). This procedure will be associated with some bleeding from the transected pancreatic surface parenchyma but can prevent the sealing of the small pancreatic duct. Bleeding can be controlled using a bipolar device or an electrocautery device.

**Figure 17:**
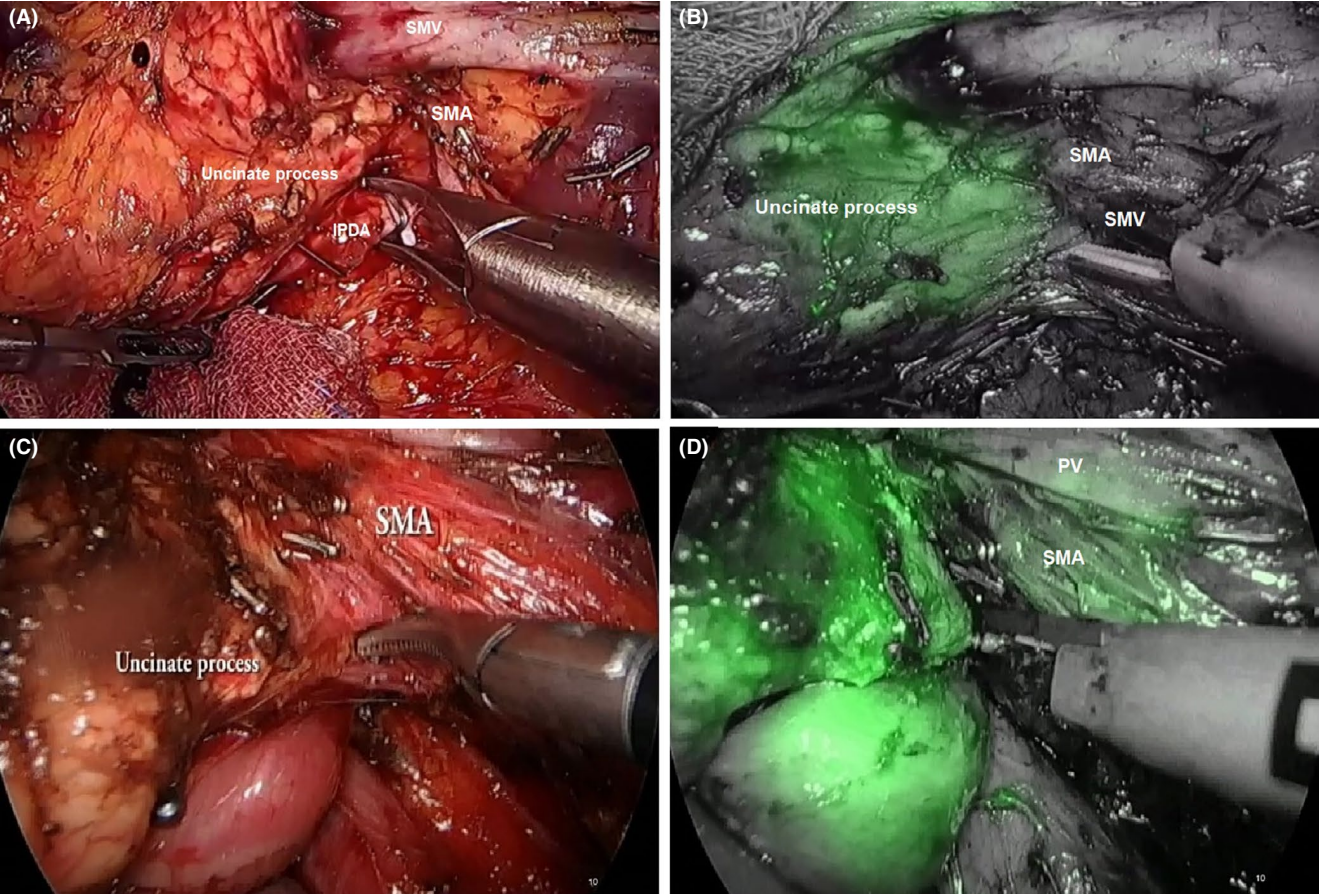
Dissection of the uncinate process (A) using indocyanine green (ICG) (B). C, Laparoscopic clips effectively secure the feeding vessels from the superior mesenteric artery (SMA) to the pancreatic head. D, Dissection of the lateral wall of the SMA using ICG. Note that ICG perfusion in the uncinate process can be differentiated from the SMA lateral border. IPDA, inferior pancreaticoduodenal artery; PV, portal vein; SMV, superior mesenteric vein

Once the pancreas is transected, dissection of the uncinate process is carried out. Complete exposure of the uncinate process can be accomplished by superior‐medial retraction of the transverse mesocolon. This retraction can also clearly define the border of the uncinate process of the pancreas. It is important to emphasize that there are multiple small vessels originating from the SMV, PV, and SMA between the connective tissues of these vessels and the uncinate process and the head of the pancreas. Dissection can be facilitated using an energy device and laparoscopic clips. With the magnified view of the laparoscope, small vessels can be easily recognized and safely clipped. However, dissection of the uncinate process is among one of the most difficult parts of the resection stage. In particular, the inferior pancreaticoduodenal artery (IPDA) from the SMA can be encountered on the posterior border of the SMV (Figure 17A). This artery should be ligated using laparoscopic clips, and dissection is continued along the lateral border of the SMA. Likewise, ICG used during this dissection can clearly define the border of the uncinate process and the retroperitoneal resection margin of dissection^33^ (Figure 17B‐D). The left‐hand grasper retracts the SMV superomedially to expose the soft tissues along the lateral border of the SMA. All connective tissues along the lateral wall of the SMA are dissected using a harmonic device. Small vessels can be secured with laparoscopic clips.

#### 5.1.13 Division of the bile duct

**Figure 18:**
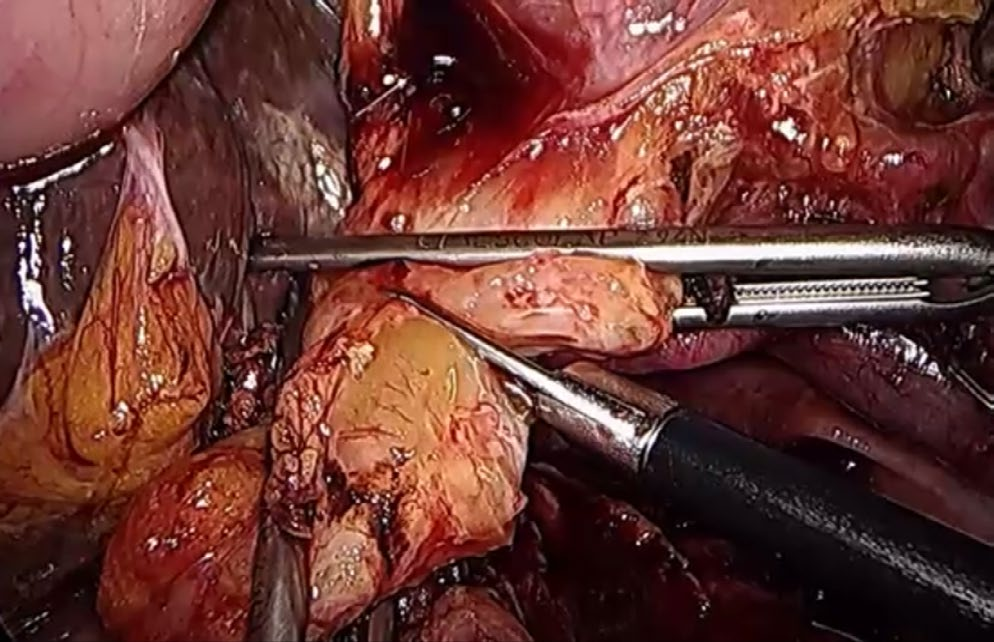
Transection of the common hepatic duct

After the head of the pancreas is free, the proximal portion of the common hepatic duct is clipped with a bulldog clamp to avoid bile spillage after transection while the distal portion is clipped with a hemoclip or bulldog clamp. The common hepatic duct is transected just above the origin of the cystic duct using scissors (Figure 18). The specimen is placed into an endo‐pouch. Cholecystectomy will be the last step in the resection phase for effective liver retraction to the end of the resection phase.

### 5.2 Reconstruction stage

This stage is believed to be one of the biggest obstacles to wide application of Lap PD in clinical practice. However, when surgeons know the basic surgical principles for pancreaticojejunostomy (PJ) and hepaticojejunostomy (HJ) and some technical tips, these procedures can be safely carried out. In the near future, robotic surgical systems will play a large role in this stage. However, as a result of its high cost, not every patient can undergo robotic surgery. Therefore, pancreatic surgeons must have good surgical skills to undertake these challenging surgical procedures to fulfill the goal of minimally invasive surgery.

#### 5.2.1 Managing remnant pancreas

This step is the most challenging. However, through didactics and simulation, this step can be safely conducted. Instruments must be handled carefully to avoid cutting and injury to the pancreatic remnant during the anastomosis. Usually, four stitches are applied for a <2‐mm pancreatic duct‐to‐mucosa PJ. For a duct >2 mm, six or more sutures can be used depending on the size of the duct. Achieving an exact angle of the needle is one of the most difficult steps during anastomosis. The following steps are our surgical strategy in managing the remnant pancreas during Lap PD.

1. Interrupted suture, two‐layer, duct‐to‐mucosa PJ with a short stent is our standard approach for managing the remnant pancreas.
2. A laparoscopic camera is placed through Port B, using Port a and Port A as working ports to allow a more ergonomic position.
3. The jejunal limb is brought to the pancreatic stump in a retrocolic way. In specific cases, the jejunal limb can be pulled up through the retromesenteric window.
4. The posterior layer is sutured first from the superior to the inferior border of the pancreas. After reaching the pancreatic duct, stay sutures are used in the pancreatic duct. A 6 o'clock suture is placed before completing the outer/posterior interrupted sutures. Usually, one to two stay sutures in the posterior aspect of the duct (6 o'clock position) are used to avoid suture confusion.
5. The posterior layer interrupted sutures are continued.
6. Next, the duct‐to‐mucosa anastomosis is continued. Stay sutures in the 3 and 9 o'clock positions are used at the jejunal opening. Additional sutures in the 12 o'clock position are used depending on the size of the pancreatic duct.
7. Once the duct‐to‐mucosa anastomosis is achieved, the anterior layer is completed using interrupted sutures from superior to inferior border pancreas.

**Figure 19:**
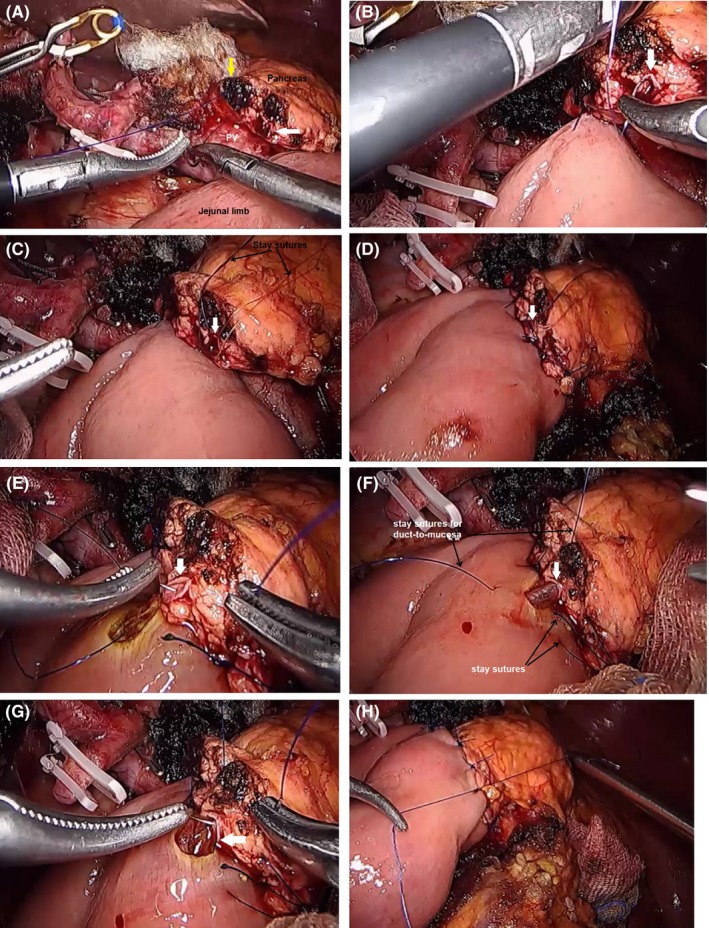
Pancreaticojejunostomy anastomosis. A, Simple interrupted suture of prolene 5‐0 from the superior to inferior pancreas (yellow arrow). B‐C, After one or two sutures, two stay sutures in the 6 o'clock and 9 o'clock positions of the pancreatic duct are usually placed before completing the posterior layer sutures. D, Completed posterior layer sutures. E, Completion of the duct‐to‐mucosa anastomosis with a 3 o'clock suture. F, Placement of a stent. G, The 12 o'clock duct‐to‐mucosa suture. H, Completed pancreaticoduodenal anastomosis. White arrow, pancreatic duct; PV, portal vein

Rotation with a blunt angle‐fashioned needle mounted on a laparoscopic needle holder is carried out allowing a curving (spiral) movement for appropriate PJ suturing. Figure 19 shows the steps in pancreaticoduodenal anastomosis.

#### 5.2.2 Managing bile duct reconstruction

**Figure 20:**
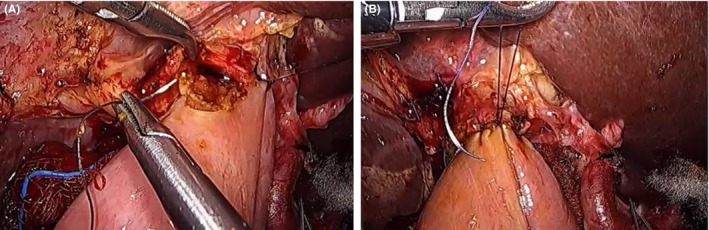
Hepaticojejunostomy anastomosis. A, Continuous running suture (Vicryl 5‐0, Ethicon Inc., Johnson and Jonson Medical, Somerville, NJ, USA) on the posterior side. B, Simple interrupted sutures (Vicryl 5‐0) on the anterior side

Posterior continuous and anterior interrupted sutures are placed for hepaticojejunostomy (Figure 20). Using different types of suture materials; for example, multifilament absorbable sutures and monofilament absorbable/non‐absorbable sutures, makes it much easier to differentiate between two stitches in a narrow surgical space. Tension should be carefully checked to avoid disruption of the anastomosis and bile leak. We usually use a 15‐cm jejunal limb from the PJ site to avoid tension.

#### 5.2.3 Duodenojejunostomy

**Figure 21:**
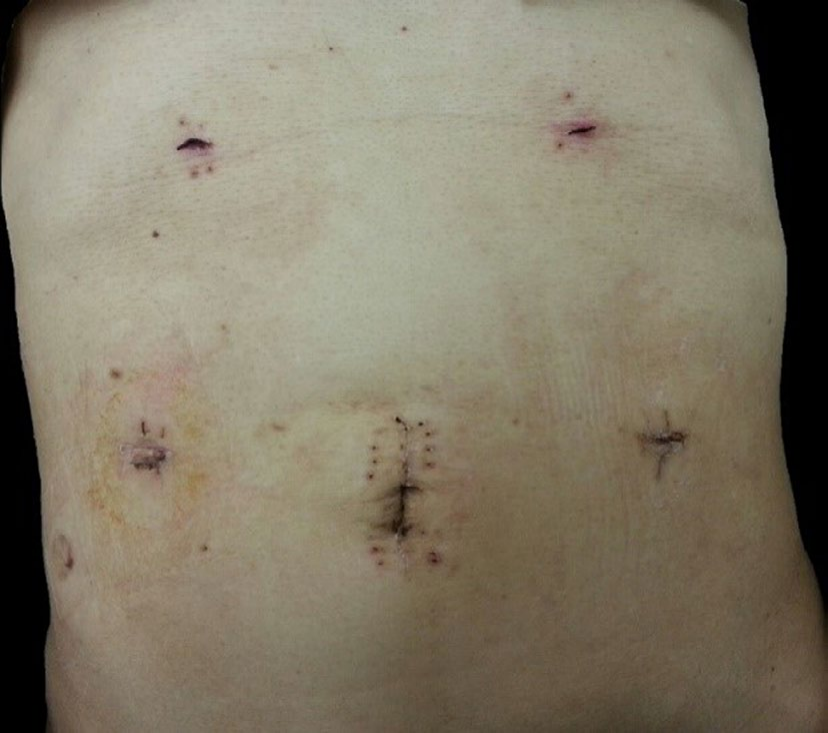
Postoperative wound after laparoscopic pancreaticoduodenectomy

The umbilical wound (Port‐A site) is extended by approximately 5 cm, which is enough to accommodate extraction of the specimen. A 50‐cm Roux‐en‐Y limb of the jejunum from the hepaticojejunostomy site is marked for the duodenojejunostomy. The duodenum and jejunum must be carefully oriented laparoscopically, tagged with sutures, and removed through the small umbilical wound for anastomosis. End‐to‐side or side‐to‐side duodenojejunostomy can be carried out manually. Figure 21 shows the postoperative wound after Lap PD.

## 6 MERGING ICG TECHNOLOGY IN PANCREATIC SURGERY

As a preliminary form of image‐guided surgery, intraoperative ICG can help in deciding whether to carry out minimally invasive pancreatectomy. Kim et al^36^ reported that, because the pancreas is a well‐vascularized organ, intravenous ICG will accumulate in the pancreas parenchyma. As such, they documented its usefulness in distal pancreatectomy to clearly define the appropriate margin of resection in a case of solid pseudopapillary neoplasm with poor uptake of ICG.

In Lap PD, initially reported by Rho et al,^34^ ICG can identify adequate perfusion of the pancreas at the pancreaticojejunostomy anastomotic site by measurement of tissue fluorescence after injection during Lap PD. ICG was then used, in certain circumstances, to determine the appropriate surgical plane for dissecting the retroperitoneal margin, as it can be well identified with ICG technology.^33^ However, further studies need to be undertaken to prove this usefulness. Nevertheless, since then, our center has used ICG as an adjunct to properly identify the plane of dissection in the uncinate process of the pancreas and on the lateral margin of the SMA.

## 7 SUMMARY OF RETROSPECTIVE STUDIES OF THE FEASIBILITY AND SAFETY OF LAP PD

Recently, a number of retrospective studies evaluating the efficacy of Lap PD have been carried out^21^, ^37^, ^38^, ^39^, ^40^, ^41^ (Table 2). These studies included more than 50 cases of Lap PD in a single‐institution series. Operative time ranged from 220 minutes to 810 minutes, with operative blood loss ranging from 45 mL to 8500 mL. Length of hospital stay ranged from 4 to 69 days depending on the associated morbidity after surgery. Vascular resections were also carried out.^37^ In addition, the rate of pancreatic fistula was 10.1%. The rate of conversion to open PD was 2.9%. R0 resection rate ranged from 84% to 100%. All of the studies concluded that Lap PD is safe and feasible, especially when carried out in a high‐volume center by experienced surgeons. Moreover, the Lap PD procedure in these case series was done by well‐experienced and expert surgeons in the field of pancreatic surgery. More importantly, these surgeons were able to standardize their approach, perhaps as a result of their cumulative experience in laparoscopic surgery.

**Table 2:** Summary of retrospective studies of Lap PD in a single‐institution series

Author/Year	No. of patients	Operation time	Blood loss	LOH	Morbidity N (%)	Mortality	R0	Conversion rate
Kendrick & Cusati^21^ (2010)	Retrospective cohort 62 59‐PD 8‐Robot assisted	368 (median)	240 mL (median)	7 (4‐69)	N = 26 (41.9) POPF = 11 (17.7) DGE = 9 Bleeding = 5 DVT = 2	1	89%	2
Duan et al^38^ (2017)	Retrospective cohort 57	315 (220‐575)	200 (100‐550)	14.8 (8‐29) (mean)	N = 28.1% POPF B = 5.3% Bleeding = 7% DGE = 1.8% Intra‐abdominal infection = 5.3% Pulmonary infection = 5.3% Ileus = 1.8% 3 reoperations	0	100%	3
Palanisamy et al^39^ (2015)	Retrospective cohort 130 Lap Classical Whipple = 65 Lap PD = 85	310 ± 34	110 ± 22	8 ± 2.6	N = 29.7% POPF B = 4.62% C = 3.84% Bleeding B = 0.76% C = 1.53% DGE A = 5.38% B = 3.07% C = 2.31% Cardiac = 2.3% Pulmonary = 10.76% Wound infection = 3.84% Intra‐abdominal abscess = 1.5% Reoperation = 3.84%, 5	2	90.8%	2
Kim et al^40^ (2013)	Retrospective cohort 105	475 (270‐810)	NR	11.5 median Range = (7‐40)	N = 25 POPF B/C = 6 (6%) Ileus = 5 (5) Bleeding = 5 Bile leak = 3 Marginal ulcer = 1 Chyle leak = 2, 2 Wound infection = 1 DGE = 2, 2 Wound dehiscence = 1	0	88%	NR
Delitto et al^41^ (2016)	Comparative retrospective study Lap PD = 52 Open PD = 50	361 ± 6	260 ± 36	9 ± 0.7 mean	Clavien‐Dindo III/IV = 13 (25%) Pancreatic leak Grade B/C = 6 (12%) Hemorrhage = 5 (10%)	1	91%	9%
Stauffer et al^37^ (2017)	Comparative retrospective study Lap PD = 58 Open PD = 193	518 (313‐761)	250 (50‐8500	6 (4‐48)	Clavien‐Dindo III‐IV = 13 Wound infection = 5 (8.6%) DGE A = 4 (6.9%) B = 3 (5.2%) C = 3 (5.2%) Hemorrhage A = 1 (1.7%) B = 1 (1.7%) C = 2 (3.4%) POPF = 6 (1.8%) A = 2 (3.9%) B = 4 (7.8%)	3.4%	84.5%	NR

DGE, delayed gastric emptying; DVT, deep vein thrombosis; Lap PD, laparoscopic pancreaticoduodenectomy; LOH, length of hospital stay; NR, not reported; Open PD; open pancreaticoduodenectomy; POPF, postoperative pancreatic fistula; R0, resection margin negative.

## 8 CONCLUSION

Although open PD remains the standard treatment for benign and malignant periampullary tumors, it is cautiously thought that Lap PD can be a good alternative option for well‐selected patients with experienced laparoscopic pancreatic surgeons. The innate complexity of the procedure itself is the main pitfall of Lap PD. However, for Lap PD to become accepted as safe and feasible in general, standardization of the procedure is paramount. Our collaborative strategy of outside‐the‐operating room didactics and simulation and inside‐the‐operating room step‐by‐step mastery of the lap PD procedure may considerably overcome the volume‐based learning curve of lap PD.

## DISCLOSURE

Conflicts of Interest: Authors declare no conflicts of interest for this article.
